# Complete Genome Sequence of Lactococcus lactis subsp. *lactis* bv. diacetylactis SD96

**DOI:** 10.1128/MRA.01140-19

**Published:** 2020-01-16

**Authors:** Robin Dorau, Jun Chen, Peter Ruhdal Jensen, Christian Solem

**Affiliations:** a National Food Institute, Technical University of Denmark, Kongens Lyngby, Denmark; University of Arizona

## Abstract

The genome of Lactococcus lactis subsp. *lactis* bv. diacetylactis SD96, a strain used for cheese production, is presented. SD96 is refractory to phage attack, which is a desired property for starter bacteria. Its 10 plasmids provide industrially important traits, such as lactose and citrate metabolism, proteolytic activity, and phage resistance.

## ANNOUNCEMENT

Lactococcus lactis, considered to be the model organism of lactic acid bacteria, has a long history of use in the dairy industry. Many mesophilic starter cultures used for cheese production contain several lactic acid bacteria, which are usually *Lactococcus* and *Leuconostoc* species (1). Lactococcus lactis subsp. *lactis* bv. diacetylactis is present in many mesophilic starter cultures and is responsible for a buttery aroma and gas formation from citrate. It has been shown that large genetic variety exists among lactococci, even between members of the same subspecies (2, 3).

Here, we present the whole genome of L. lactis subsp. *lactis* bv. diacetylactis SD96, a bacteriophage-insensitive mutant of strain SD16, which was originally isolated from an Italian cheese by starter culture provider Sacco S.r.l. To date, none of the phages contained in the comprehensive phage collection at Sacco S.r.l. has been found to be able to infect SD96, and dairies using SD96 have not reported any phage attack for this strain. Because of the contributions of plasmid-encoded genes to industrially relevant phenotypes, we were particularly interested in the plasmid content of this strain.

DNA for sequencing was isolated from an early-stationary-phase culture in M17 broth supplemented with 0.5% lactose, which was inoculated from a single colony. Total DNA was isolated using a genomic DNA purification kit (product number K0512) from Thermo Scientific. Sequencing was carried out on both PacBio Sequel and Illumina HiSeq 4000 platforms, using two distinct DNA preparations. Illumina sequencing using a PE150 PCR-free library provided 8.78 million raw paired-end 150-bp reads (SRA accession number SRX6686433). The sequences were processed and assembled using various software programs with default settings unless otherwise stated. Raw Illumina reads were processed using Geneious Prime (version 2019.1.3) (4). Briefly, adapter sequences and low-quality sequences were trimmed from both ends; finally, all reads above 50 bp were kept. This process resulted in 7.25 million sequences, with an average length of 138 bp. From the PacBio Sequel project using a 20-kb library, we received 331,855 raw reads, with an average length of 7,575 bp and a maximal read length of 44,814 bp (SRA accession number SRX6711783). For *de novo* assembly, the PacBio reads alone were processed and assembled using the Canu assembler (version 1.8) (5), which provided 13 linear scaffolds. The Circlator tool (version 1.5.5) (6) provided a closed chromosome and four plasmids. Another six plasmids were closed by identifying repeating regions on the scaffolds using the Repeat Finder plugin (version 1.0) in Geneious, followed by manual evaluation and trimming. Furthermore, the presence of replication-associated genes was confirmed for all 10 plasmids by sequence comparison with previously characterized L. lactis plasmids. Two scaffolds (4,944 and 5,967 bp) could not be closed or associated with the genome and did not contain replication-associated genes. Finally, the circularized sequences were polished using Pilon (version 1.23), for which the processed Illumina paired-end reads had been mapped to the circular sequences using Bowtie2 (version 2.3.0) in Geneious. The genome and plasmids were annotated using the RAST server (version 2.0) (7).

The 2.42-Mbp chromosome of SD96 has a GC content of 35.3%. The 10 plasmids have an average GC content of 34.4% (minimum, 30.3%; maximum, 36.5%) and sizes of 64.9, 49.6, 37.4, 25.4, 11.7, 8.3, 7.9, 7.3, 4.6, and 2.7 kbp (GenBank accession numbers CP043518 to CP043528). Plasmids associated with protein degradation, lactose and citrate metabolism, and phage defense, as well as two cryptic plasmids with unknown purpose, were identified.

### Data availability.

The genome sequences of L. lactis subsp. *lactis* biovar diacetylactis SD96, including its 10 plasmids, are available in GenBank under accession numbers CP043523 (chromosome), CP043525 (pSD96_01), CP043526 (pSD96_02), CP043527 (pSD96_03), CP043528 (pSD96_04), CP043524 (pSD96_05), CP043518 (pSD96_06), CP043519 (pSD96_07), CP043520 (pSD96_08), CP043521 (pSD96_09), and CP043522 (pSD96_10). Raw data have been deposited in the Sequence Read Archive (SRA) under accession numbers SRX6686433 (Illumina data) and SRX6711783 (PacBio Sequel data).
